# Biomining of lunar regolith simulant EAC-1 A with the fungus *Penicillium simplicissimum*

**DOI:** 10.1186/s40694-025-00201-z

**Published:** 2025-05-19

**Authors:** João Figueira, Stella Koch, Daniel W. Müller, Sebastian Slawik, Aidan Cowley, Ralf Moeller, Marta Cortesão

**Affiliations:** 1 Institute of Aerospace Medicine, Radiation Biology Department, Aerospace Microbiology Research Group, German Aerospace Center (DLR), Cologne, Germany; 2 Department of Biology, Faculty of Sciences, University of Porto (FCUP), Porto, Portugal; 3 Spaceship EAC, European Space Agency (ESA), Cologne, Germany; 4 Chair of Functional Materials, Department of Material Science, Saarland University, Saarbrücken, Germany

**Keywords:** ISRU_1_, biomining_2_, moon_3_, Fungal biotechnology_4_, penicillium_5_, Lunar regolith_6_, bioleaching_7_, Space exploration_8_, Lunar habitat_9_, LUNA_10_

## Abstract

**Background:**

On a future lunar habitat, acquiring needed resources in situ will inevitably come from the Lunar regolith. Biomining, i.e. the use of microorganisms to extract metals from the regolith, is sustainable and energy-efficient, making it highly promising for space exploration applications. Given the extensive use of filamentous fungi in industrial biotechnology, we investigated the ability of the fungus *Penicillium simplicissimum* to extract metals from the European Astronaut Centre lunar regolith simulant 1 (EAC-1 A), which will be used as the analogue soil at the European Lunar Exploration Laboratory (LUNA) facility at the European Space Agency (ESA) and German Aerospace Centre (DLR) site.

**Results:**

Biocompatibility tests demonstrated *P. simplicissimum* tolerance to high concentrations of EAC-1 A lunar regolith simulant (up to 60%), both on Earth gravity and Lunar simulated gravity via clinorotation. We reveal that a fungal bioleaching setup using low nutrient medium (20% PDB) enables *P. simplicissimum* to extract metals from EAC-1 A regolith over the course of 2 weeks at room temperature. Inductively coupled plasma mass spectrometry (ICP-MS) analysis of the leachate revealed the extraction of magnesium (up to 159 mg/L), calcium (151 mg/L), iron (68 mg/L), aluminium (32 mg/L), manganese (3 mg/L) as well as traces of titanium (0.02 mg/L). The recovered metal oxide powder from the leachate, obtained via centrifugation (14,500 g, 4,000 rpm), followed by filtration (0.22 μm) and drying at 60 °C overnight, achieved a promising average of 10 ± 3 g/L. Further analysis via SEM/EDS and XRD confirmed the presence of aluminium [as boehmite (AlO(OH))], magnesium, and iron [possibly as haematite (Fe_2_O_3_)] and magnetite [possibly as (Fe_3_O_4_)].

**Conclusion:**

Our study demonstrates successful fungal biomining of lunar regolith simulant EAC-1 A and emphasizes the utilization of fungal-based approaches as promising ISRU technologies in future space exploration missions.

**Supplementary Information:**

The online version contains supplementary material available at 10.1186/s40694-025-00201-z.

## Introduction

In future lunar exploration missions, transporting resources from Earth will be one of the main challenges [1]. To reduce the logistical challenge of resupply using Earth’s resources, In situ Resource Utilization (ISRU) approaches will play a critical role in obtaining essential goods in a cost-effective and sustainable manner [2, 3]. In a Lunar exploration scenario, raw materials such as the soil, are widely available and can be explored for use in various areas, from life support systems (water-H_2_O) to fuels and propellants (hydrogen-H_2_) [4]. In particular, the Lunar regolith - a superficial layer of unconsolidated materials – is expected to be a primary source of in situ resources, mainly through mining and extraction technologies [5]. Samples recovered from Apollo’s missions reveal that Lunar regolith comprises a vast variety of metals (Table 1), for example, iron, aluminium, or titanium [6]. These metals can be mined in situ and be used for the fabrication of equipment, construction materials, as well as to integrate devices for power generation [7]. Among the various power generation technologies available, solar cells stand out as a promising option for lunar exploration, as they can be produced entirely in situ using highly abundant materials such as silicon, aluminium, and other metals found in lunar regolith [8].

To prepare astronauts for human exploration of the Moon, the European Space Agency (ESA) and the German Aerospace Center (DLR) are currently constructing the European Lunar Exploration Laboratory (LUNA), in Cologne, Germany. The LUNA facility will work as a testbed to explore the technologies that might ease human settlements beyond Earth. To fill LUNA’s 660 m^2^ area with lunar regolith simulant material, the European Astronaut Centre lunar regolith simulant 1 (EAC-1 A) - a basaltic sandy silt from a quarry located in the Siebengebirge volcanic field - is considered as a large-volume source of material [9]. EAC-1 A is comparable with widely used lunar regolith simulants, namely JSC-1 A, JSC-2A, NU-LHT-3 M, DNA and FJS-1, each with slightly varying properties such as the grain size or composition [9–11].

**Table 1:** Abundance of chemical compounds with metallic elements from the sample 70,017 recovered from the Moon during the mission Apollo 17, and from EAC-1 A lunar regolith simulant, adapted from [6, 9]

COMPOUND	ABUNDANCE FROM APOLLO 17 (wt%)	ABUNDANCE FROM CHANG’E-5 (wt%)	ABUNDANCE IN EAC-1 A (wt%)
SiO_2_	38.37	42.2	44.35
FeO	18.71	22.5	11.35
TiO_2_	12.83	5.0	2.4
CaO	10.43	11.0	10.8
MgO	9.41	6.5	11.9
Al_2_O_3_	8.78	10.8	12.6
Na_2_O	0.43	0.26	2.9
MnO	0.28	0.28	11.9

While mining is fundamental for our civilization, traditional methods are undesirable for sustainable space exploration, due to high energetic costs and environmental damages [12]. Alternative mining approaches such as molten salt electrolysis or the FFC process, are currently being studied to liberate metals from the lunar regolith, however these processes are still energetically intensive [13]. In contrast, the use of microorganisms to extract desirable metals, i.e. biomining, is a sustainable and energy-efficient biotechnological alternative to obtain metals for human space exploration [14]. On Earth, biomining is responsible for up to 25% of copper and 5% of the total gold mining [15] and can be used in the recovery of metallic elements from electronic waste [16].

Filamentous fungi are notably known for their role in bioleaching: a biomining method that mobilizes the metals from the ores into the liquid culture medium. This happens as filamentous fungi excel at producing and excreting organic acids, performing an organic acid-mediated bioleaching. The fungus *Penicillium simplicissimum* is currently used to biomine copper and has been reported to effectively mine valuable components from electronic waste [17]. *P. simplicissimum* bioleaches via its natural production of organic acids, such as citric or oxalic acid. These organic acids are secreted into the surrounding medium where they interact with the minerals, breaking down chemical bonds [18]. As a direct result from the accumulation of the organic acids in solution, the pH lowers, which increases the solubility of most metals and promotes the release (or leaching) of metals into the leachate solution [14]. This process is called acidolysis, which is dominant in fungal bioleaching, although other biomechanical and chemical processes can simultaneously take place to help mobilize metals from the regolith being bioleached.

Given the wide use of filamentous fungi on Earth’s industrial biotechnology (from antibiotic production to food preservation or cosmetics) fungal biomining in space can be a promising ISRU technology, to provide the crew with metals for construction pipelines or energy generation, as well as to aid waste-management through fungal biodegradation and bioremediation [19–24]. From a logistical perspective, the implementation of fungal bioleaching can be easily carried to space, as fungal spores are dormant resistant structures and are known to be highly resistant to space radiation, making them favourable players for transport and stowage during space travel [22, 25]. Besides, filamentous fungi *Aspergillus* spp. and *Penicillium* spp. can grow outside Earth’s gravitational conditions, as they are commonly identified as part of the microbiome of space stations in Low Earth Orbit (LEO), such as Mir and the International Space Station (ISS) [22, 25, 26] and will likely continue to be present in future crewed long-term missions to the Moon.

A different gravitational regime on the Moon creates the necessity of fully comprehending the effect of gravity, not only on humans, but also on technological and microbial processes to be carried out in situ [27]. Space biomining has been successfully tested aboard the International Space Station, demonstrating the viability of using bacteria, such as *Bacillus subtilis* and *Sphingomonas desiccabilis*, to extract rare-earth elements [28]. *S. desiccabilis* showed no significant differences in metal mobilization in response to different gravitational regimes, however the biomining potential of *B. subtilis* was reduced. A summary of microbial biominining studies in the context of space exploration can be found in Table 2. Recent developments in space biomining have been exclusive to bacterial species, as demonstrated in Table 2. However, fungi play a dominant role in terrestrial biomining due to their ability to produce and secrete organic acids and other metabolites that facilitate metal leaching. Given their resilience, ease of spore storage, and metabolic versatility, fungi could offer significant advantages over bacteria in space applications. Despite this, there is a striking lack of studies on fungal biomining in space conditions. Expanding research in this direction could unlock more efficient and versatile biomining strategies for space resource utilization, bridging a critical knowledge gap in the field [29].

**Table 2:** Microbial biomining studies conducted in the context of space exploration

MICROORGANISM	SUBSTRACT	MEDIA USED	BIOMINING	OUTCOME	APPLICATION	REFERENCE
*Acidithiobacillus ferrooxidans*	Lunar Mare Simulant Mars Global Simulant	Modified 9 K medium, as detailed above	50 mL in space flight hardware under anaerobic conditions	Leaching of Si, Mn and Mg	Production of useful nanoparticles in space	[31]
*Bacillus subtilis NCIB 3610* *Sphingomonas desiccabilis CP1D*	Basalt slides as Moon and Mars rock analogs	50% R2A medium, as detailed above.	5 mL in BioRock experimental unit aboard the ISS	Leaching of Vanadium under three gravity conditions	Production of superconducting materials and batteries. Fabrication of rovers.	[32]
*Cupriavidus metallidurans*	Basaltic rock	50% R2A medium, as detailed above.	5 mL in BioRock experimental unit aboard the ISS	No significant difference compared to non-biological controls	Potential for biomining in space environments	[28]
*Penicillium simplicissimum*	Lunar regolith simulant EAC.1 A	20% (w/v) Potato Dextrose Broth (PDB) supplemented with 30% (w/v) EAC-1 A regolith simulant.	40 mL in Shake-flasks on Ground	Leaching of AluminimumAluminium, Iron, and other relevant metals	Manufacture of space relevant materials and technologies (for instance, energy generation devices)	This study
*Shewanella oneidensis*	JSC-Mars1 EAC-1 JSC-2 A	Modified 9 K medium (per liter): 0.4 g (NH₄)₂SO₄, 0.4 g MgSO₄·7 H₂O, 0.4 g KH₂PO₄, 1 mL trace element solution (with MnCl₂·2 H₂O, ZnSO₄·7 H₂O, CoCl₂·6 H₂O, H₃BO₃, Na₂MoO₄, CuCl₂·2 H₂O, NaVO)	Bacterial treatment of the regolith	Magnetic extraction of metallic elements from the regolith	Production of construction and replacement parts	[30]
*Sphingomonas desiccabilis CP1D*	Basalt slides as Moon and Mars rock analogs	50% R2A medium (per liter): 0.25 g yeast extract, 0.25 g peptone, 0.25 g casamino acids, 0.25 g glucose, 0.25 g soluble starch, 0.15 g Na-pyruvate, 0.15 g K₂HPO₄, 0.025 g MgSO₄·7 H₂O; pH 7.2.	5 mL in BioRock experimental unit aboard the ISS	Leaching of heavy Rare Earth Elements (Gd up to Lu)	Economically exploiting materials from celestial bodies	[28]

In this study we have taken the first steps to establish fungal biomining of lunar regolith by validating the use of *Penicillium simplicissimum* to extract metals from the Lunar regolith simulant EAC-1 A. In this study, we report data on biocompatibility of *P. simplicissimum* to EAC-1 A, under both Earth and simulated Lunar gravity. We demonstrate *P. simplicissimum’s* ability to mobilize non-rare metallic elements from EAC-1 A regolith into the medium solution (leachate). Additionally, we present data on fungal bioleaching culture profiling parameters (pH, total iron and organic acid levels), as well as quantification of bioleached metals by Inductively coupled plasma mass spectrometry (ICP-MS). Finally, we show the successful metal recovery from the leachate and characterize the obtained powder via SEM/EDS (Energy Dispersive X-Ray Spectroscopy) and XRD (X-Ray Diffraction).

## Materials and methods

### Media and strain

In this study, the filamentous fungus strain used was *Penicillium simplicissimum* (DSM 1097) obtained from the German Collection of Microorganisms and Cell Cultures (DSMZ). The selection of *Penicillium simplicissimum* was based on several factors. Firstly, the authors considered available data and literature regarding Earth’s biomining practices, which highlighted the resilience of certain filamentous fungi, including *P. simplicissimum*, to metal toxicity [33–35]. Additionally, *P. simplicissimum’s* significant role in current biomining practices on Earth made it a practical choice for potential space applications [15]. Furthermore, its involvement in studies related to e-waste recycling and biohydrometallurgy further supported its suitability for space applications [16]. Additionally, studies utilizing *P. simplicissimum* in space research, such as the Bioasteroid mission, contributed to its selection for the current study [14].

The fungal spores were harvested from 3 to 4 days-old cultures, grown on Potato Dextrose Agar (PDA) (Sigma), at room temperature (22–24 °C). Spore suspensions were prepared by placing 5–10 mL of saline solution (0.9% NaCl) on the agar plate and gently scraping the surface using a cotton stick. The spore suspensions were retrieved from the agar plate and then filtered with a Miracloth filter (Millipore) and stored at 4 °C. Spore concentration was determined using a Thoma counting chamber. All experiments were performed with fresh spore suspensions not older than 2 weeks. The Lunar regolith simulant used (EAC-1 A) was provided by Dr. Aidan Cowley from ESA Spaceship EAC, Cologne, Germany. Composition of EAC-1 A regolith is described by Engelschiøn et al. 2020 [9]. In all experiments utilizing medium supplemented with regolith, the regolith was previously sterilized in a powder form inside a glass petri dish or shake-flasks in a heat steriliser at 220 °C for 4 h.

### Biocompatibility tests

To test biocompatibility of the fungus with the regolith, the fungus was growth on PDA plates supplemented with different concentrations of EAC-1A [0%, 0.5%, 10%, 20%, 40% and 60% (w/v)]. Agar-medium plates supplemented with EAC-1A can be challenging due to insolubility of the regolith. Thus, to obtain EAC-1A agar plates, this study used the following method: The Lunar regolith EAC-1A powder (previously heat sterilized) was first weighted to the desired amount (to each corresponding EAC-1A concentration) using a precision scale and then transferred to a media bottle (Schott) to which was added PDA in accordance with the manufacture’ instructions. After autoclaving, the mixture (EAC-1 A and agar) was agitated, and 30 mL were collected with a 50 mL disposable sterile pipette. Due to the low solubility of EAC-1 A, the volume was collected with a serological pipette while agitating the medium to prevent sedimentation. The collected volume was then transferred to an empty, sterile petri-dish to achieve plates with a uniform regolith concentration. To test fungal growth, the plates were inoculated with 10 µL of a spore suspension with 10^6^ spores/mL for 6 days at room temperature (22–24 °C). Fungal growth was monitored via the determination of the corresponding colony area on day 2, 4 and 6. A second biocompatibility assay was performed with the same methodology, comparing fungal colony area at 0% and 60% EAC-1 A (w/v), from 0 to 9 days, with measurements on day 2, 4, 7 and 9. Determination of colony area was calculated from high resolution photographs taken with the camera Sony α-500 (APS-C), with a macro-objective (E 3.5/30). A tripod was used to ensure a constant height. Photographs taken were analysed with the Fiji/Image J software, where the colony area was measured [36].

### Growth in lunar simulated gravity (LSG)

To investigate the impact of Lunar Simulated Gravity (LSG) on the adaptive responses of the fungus *P. simplicissimum*, we conducted experiments using agar plates containing standard medium (PDA) and PDA supplemented with 60% EAC-1 A. These experiments were performed on a 2-D petri dish clinostat, a device designed to simulate altered gravity conditions. The simulation of Lunar gravity was achieved through the application of clinorotation principles, involving continuous rotation of samples at a constant speed (Allen et al., 2022). This rotation exposes the samples to a functional simulation of an altered gravity environment, centered around the rotation axis. Notably, the clinostat serves as a tool for functional simulation, preventing particle sedimentation during the experiment.

It is crucial to highlight that when positioning the colony at the central region of the plate, Lunar gravity simulation becomes discernible when the clinostat axis is set to an angle of 10° (Hasenstein and Loon, 2022). By adhering to these experimental parameters, we aim to gain insights into the adaptive mechanisms of *P. simplicissimum* under Lunar Simulated Gravity (LSG). As such, the degree to the horizontal axis of the clinostat was adjusted accordingly at a 10° angle of the rotation axis at 60 rpm, to achieve 0.16 g, corresponding to Lunar Simulated Gravity (LSG) [37–39].

Plates of the experimental LSG group were placed in the clinostat (*n* = 3), and ground control Earth gravity (1 *g*) plates were placed on the bench (*n* = 3). Both groups were incubated at room temperature (22–24 °C) for 4 days. Adaptation of the fungus to LSG was monitored after 4 days via measurements of colony area (see section above), dry biomass and spore production. Dry biomass was determined by preparing PDA plates supplemented with 0% and 60% EAC-1 A and placing an additional removable polycarbonate-filter (0.4 μm pore size) on top of the agar. This was followed by inoculation with 10 µL of fungal spore suspension, at a concentration of 10^6^ spores/mL, after which the plates were incubated at 22 °C. At the end of day 4, the filter was removed, detaching the colony from the PDA plate. Each filter carrying a colony was placed inside of a pre-weighted aluminium paper. The weight of the aluminium foil with the colony was measured before and after desiccation (dw) at 60 °C for 24 h, with a high precision analytical scale (Sartorius). Dried Biomass was calculated in accordance with the following equation:

$$\:\varvec{C}\varvec{o}\varvec{l}\varvec{o}\varvec{n}\varvec{y}\:\varvec{B}\varvec{i}\varvec{o}\varvec{m}\varvec{a}\varvec{s}\varvec{s}=\varvec{D}\varvec{r}\varvec{y}\:\:\varvec{w}\varvec{e}\varvec{i}\varvec{g}\varvec{h}\varvec{t}\:\left(\varvec{d}\varvec{w}\right)-\varvec{F}\varvec{i}\varvec{l}\varvec{t}\varvec{e}{\varvec{r}}^{\varvec{{\prime\:}}}\varvec{s}\:\:\varvec{w}\varvec{e}\varvec{i}\varvec{g}\varvec{h}\varvec{t}-\varvec{A}\varvec{l}\varvec{u}\varvec{m}\varvec{i}\varvec{n}\varvec{i}\varvec{u}\varvec{m}\:\varvec{p}\varvec{a}\varvec{p}\varvec{e}{\varvec{r}}^{\varvec{{\prime\:}}}\varvec{s}\:\:\varvec{w}\varvec{e}\varvec{i}\varvec{g}\varvec{h}\varvec{t}$$

Spore production was determined for each colony, via spore recovery followed by spore count through light microscopy, according to the protocol described in Cortesao et al. 2022 [40].

### Bioleaching verification tests

To test relevant parameters that would inform the final setup: nutrient concentration, w/v of EAC-1 A, bioreactor flasks were used, each with 40 mL volume of 100% or diluted 20% Potato Dextrose Broth (PDB) medium, to test the effect of low nutrient medium in bioleaching ability. The medium was supplemented with 0%, 30% and 60% (w/v) concentrations of Lunar regolith simulant EAC-1 A. To prepare the bioleaching cultures, the first step was to add 12–24 g the powder EAC-1 A to the bioreactor flasks, which were then submitted to sterilization in the heat-sterilizer (220 °C for 4 h). After which sterile medium, and fungal spore suspensions were added. In total, each flask was inoculated with 2.5 × 10^5^ spores (final concentration) and was incubated under agitation at 150 rpm and 22 °C for 2 weeks. The tested bioleaching cultures were monitored in the first week, by measuring medium parameters via semi-quantitative methods, according to the manufacturer’s instructions: pH (strips, Sigma), organic acids (citric acid, Megazyme, and oxalic acid kit, Sigma), and metal concentration (iron, aluminium, and calcium via semi-quantitative test strips, Sigma).

### Fungal bioleaching setup

**Fig. 1:**
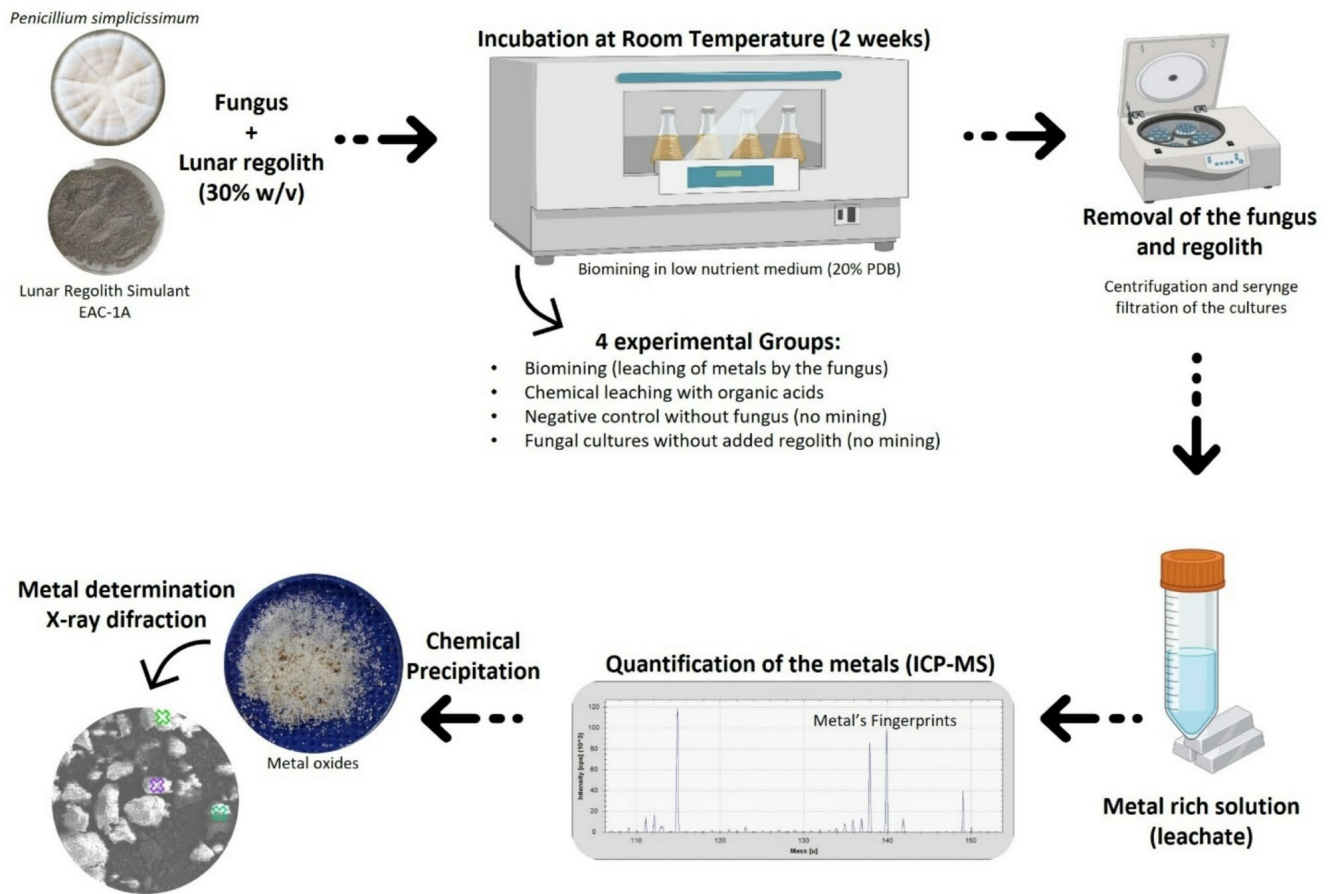
Schematic representation of the bioleaching setup and procedure for metal quantification. Bioleaching of EAC-1 A was performed over 2 weeks at room temperature and 150 rpm. Afterward, the leachate was collected and sterilized for sensitive quantification of the metal ions in solution by Inductively Coupled Plasma Mass Spectrometry (ICP-MS). The recovery of metals was achieved through chemical precipitation with sodium hydroxide, and the presence of metallic elements in the resulting metal powder oxide was validated using Energy Dispersive X-Ray Spectroscopy (EDS) and X-Ray Diffraction (XRD). The 4 experimental groups were composed of a Bioleaching culture (*Penicillium simplicissimum* spores inoculated into LN medium supplemented with 30% (w/v) EAC-1 A), chemical leaching group (LN medium supplemented with 30% (w/v) EAC-1 A and citric acid and oxalic acid), negative control (LN medium supplemented with 30% (w/v) EAC-1 A), and fungal cultures non-supplemented with EAC-1 A (*Penicillium simplicissimum* spores inoculated into LN medium). Where LN represents a medium prepared from a 20% dilution of Potato Dextrose Broth

Erlenmeyer flasks with 40 mL total volume of PDB diluted for 20% of total nutrients or non-diluted PDB (100%), each supplemented with either 0% or 30% EAC-1 A were prepared. The bioreactor flasks were inoculated with 2.5 × 10^5^ spores and kept under agitation at 150 rpm. The final bioleaching setup is schematically represented in Fig. 1. A chemical leaching control group (not inoculated) was designed with 20% PDB medium supplemented with maximum concentrations of citric acid (0.15 g/L) and oxalic acid (0.008 g/L) corresponding to bioleaching measurements at the end of 2 weeks on a bioreactor flask supplemented with EAC-1 A. A non-inoculated group of bioreactor flasks with diluted PDB (20%) with 30% EAC-1 A (w/v), and diluted PDB (at 20%, and with 0% EAC-1 A) were used as blanks for comparison. The bioleaching process was monitored via measurement of organic acid production: citric acid (K-CITR kit, Megazyme) and oxalic acid (Kit, Sigma-Aldrich) levels were measured according to the manufacturer’s instructions, in 96-well plate assays, over the first week (on days 2, 3, 4 and 7). By the end of 2 weeks, the biomass and the regolith were removed by centrifugation at 14 500 g (4000 rpm) for 30 min to isolate the leachate. The samples were then sterilized by filtration with a Whatman paper filter of pore size 0.2 μm before adding the samples, 15 mL falcon tubes were washed with MilliQ water to prevent chemical contamination. The solutions were kept at 4 °C until shipped for precise detection and quantification of the metal ions by ICP-MS (service of Medizinisches Labor, Bremen, Germany).

### Metal recovery and spectroscopy/phase analysis

Recovery of the metals from the leachate was performed by selective chemical precipitation (*n* = 3). This process precipitates the metals by adjusting the pH progressively to 10 with NaOH and HCl (both elements detected in the XRD results). Separation of the metals of interest from the leachate was done with cycles of centrifugation at 14 500 *g* (4000 rpm) for 20 min, due to their partial solubilities. The collected metals were then dried at 60 °C overnight, resulting in a final precipitate’s powder. The metallic elements of the resulting powder were initially confirmed by re-solubilization and measurement by the semi-quantitative methods described. Analysis of the powder content provided information on the chemical nature of the recovered metals. Precipitate’s analysis included Scanning Electron Microscopy and Energy Dispersive X-Ray Spectroscopy (SEM/EDS) to validate the metallic content of the samples and XRD to confirm spectroscopic analysis and detect relevant crystallographic phases. SEM was conducted on a dual beam workstation (*Helios Nanolab 600* by *FEI*) including the system´s EDS module. SEM imaging and EDS was conducted in secondary electron (SE) contrast mode at an acceleration voltage of 15 kV and a current of 1.4 nA. To allow for analyzation, the powder samples were collected on adhesive carbon patches, while a ~ 10 nm layer of Pd was applied via sputter coating prior to analysis to improve sample conductivity. X-Ray Diffraction (XRD) was performed on a *PANalytical Empyrean* diffractometer using Bragg-Brentano geometry and a low background silicon single crystal sample holder plate. On primary side a copper tube and low background optics (BBHD) and fixed slits were used, whereas the secondary beam path contained a fixed anti-scatter slit with semiconductor detector PIXcel3d in 1D channel mode.

### Statistical analysis

Data analysis and graphical representation were performed using SigmaPlot 14.5 software. All experiments were conducted with at least *n* = 3 replicates, and the graphs displayed here show the mean and standard error. Statistical analysis was performed using the Kruskal-Wallis test for non-parametric datasets (biocompatibility − 9 days) and Analysis of Variance (ANOVA) for parametric datasets (biocompatibility − 6 days), after passing the Shapiro-Wilk (normality test) and Brown-Forsythe (equal variance test). In all cases, statistical significance was considered when *p*-value < 0.05. The statistical differences in biocompatibility under Lunar Simulated Gravity (LSG) were determined using ANOVA for the parametric dataset (colony area, biomass, and spore production), which also passed the Shapiro-Wilk (normality test) and Brown-Forsythe (equal variance test).

## Results

Biomining of lunar regolith has the potential to provide future crews with useful metals produced in situ, in a cost-effective and sustainable way. In this study, we investigated biomining of EAC-1 A Lunar regolith simulant using the filamentous fungus *P. simplicissimum*. We first assessed the fungus’ tolerance to EAC-1 A, in both Earth and Lunar simulated gravity conditions. We then performed bioleaching verification tests to evaluate the fungus’s ability to extract non-rare metallic elements from the Lunar regolith into culture medium (leachate). Finally, we established a fungal bioleaching setup that enabled recovery of several metals in powder (Fig. 1).

### Biocompatibility with EAC-1 A lunar regolith simulant

**Fig. 2:**
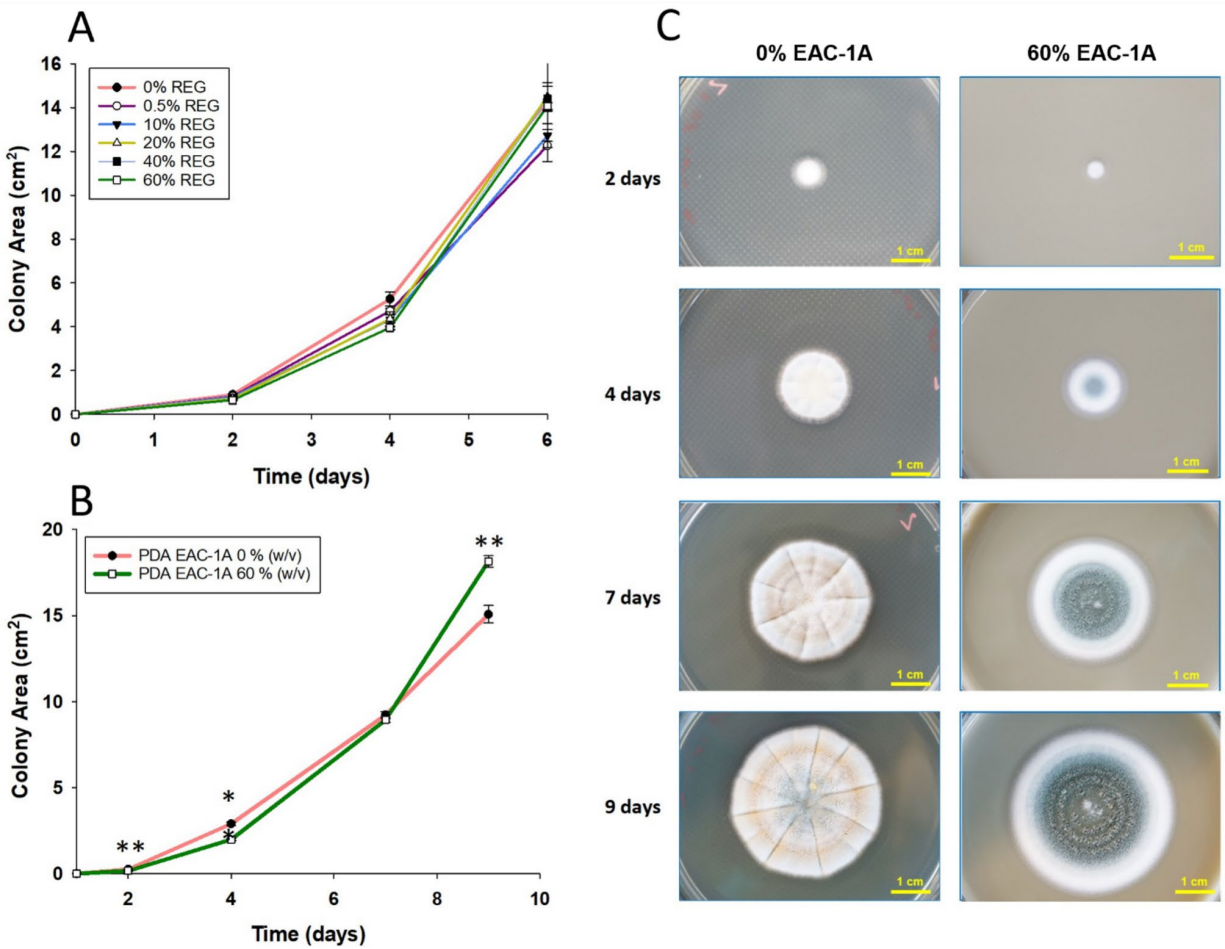
Biocompatibility of the fungus *P. simplicissimum* to EAC-1 A lunar regolith simulant **(A)** Colony area of fungus grown in PDA (Potato Dextrose Agar) medium supplemented with 0%, 0.5%, 10%, 20%, 40% and 60% (w/v) of EAC-1 A regolith, over 6 days (*n* = 3 per group). **(B)** Colony area of fungus grown in PDA medium supplemented with 0%, and 60% (w/v) of EAC-1 A regolith, over 9 days (*n* = 8 per group). **(C)** Colony morphology of *P. simplicissimum* grown in PDA medium with 60% EAC-1 A regolith (left), and without EAC-1 A (right). Yellow bar corresponds to 1 cm. ** represents *p* < 0.001 (Kruskal-Wallis test)

Given the potential toxicity of the Lunar regolith and its simulants [41–43] we evaluated the biocompatibility of EAC-1 A to *P. simplicissimum*, monitoring colony area under increasing concentrations of Lunar regolith of 0%, 0.5%, 10%, 20%, 40% and 60% (Fig. 2). Results show a decrease in colony area when the fungus was exposed to the highest concentration of EAC-1 A (60%) at day 2 (*p* < 0.001) and day 4 (*p* < 0.001), suggesting a stress response of the fungus to the regolith simulant (Fig. 2A). However, by day 6, no difference was detected in fungal colony area when grown with lunar regolith (*p* = 0.2), suggesting an adaptation of the fungus to the supplementation of EAC-1 A over time. A second run of biocompatibility (*n* = 8) was performed, comparing growth at 0% and 60% EAC-1 A (w/v) over a longer incubation period of 9 days (Fig. 2B-C). Results show that when grown in 60% EAC-1 A, the colony area of the fungus was significantly smaller by 35% at day 2, and smaller by 31% at day 4, (*p* < 0.001). However, on day 9, supplementation with EAC-1 A 60% registered a 20% increase in colony area (*p* < 0.001), again suggesting an adaptation of the fungus to the supplementation of EAC-1 A over time. Slight differences in colony area between the two experiments likely result from fluctuations in humidity, light, and room temperature. Supplementation with 60% EAC-1 A regolith also impacted fungal colony morphology, displaying differences in vegetative growth (characterized by the white colour of hyphal cells) and spore physiology (evidenced by the green colour of spores’ pigmented cell walls) (Fig. 2C). Overall, our finding suggested that exposure to Lunar regolith EAC-1 A did not prevent growth or significantly impacted colony area of *P. simplicissimum* from day 4 onwards.

### Biocompatibility under Lunar Simulated Gravity (LSG)

**Fig. 3:**
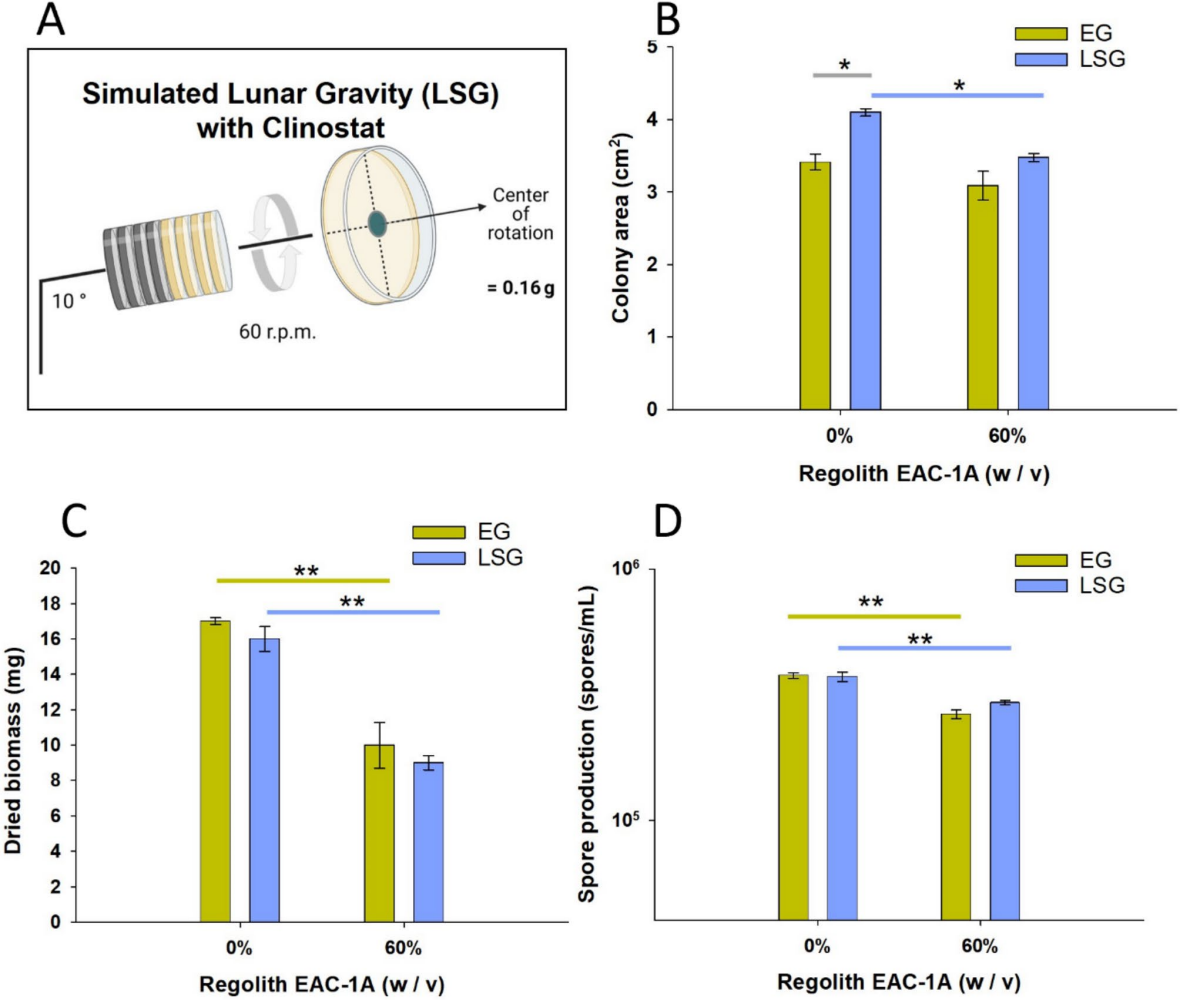
Impact of simulated Lunar gravity (LSG) on *P. simplicissimum* growth parameters when grown in agar plates with and without EAC-1 A, over 4 days. **(A)** Set-up of 2-D petri dish clinostat with rotation axis set at 10° angle to simulate Lunar Gravity (LSG). **(B)** Colony area of the fungus in exposure to EG and LSG, when supplemented with 0 or 60% EAC-1 A, suggesting a slight increase of the colony area when exposed to LGS and a significant decrease on the colony area when supplemented with EAC-1 A under Earth’s gravity (*n* = 3 per group). **(C)** Dried biomass of the fungus in exposure to EG and LSG, when supplemented with EAC-1 A suggesting a decrease caused by the supplementation of the medium with EAC-1 A in both gravitational regimes (*n* = 3 per group). **(D)** Spore production in exposure to EG and LSG, when supplemented with 60% EAC-1 A suggesting regolith leads to less spore production both EG and LSG (*n* = 3 per group). Where * represents *p* < 0.05, and ** *p* < 0.001 (One Way ANOVA)

**Fig. 4:**
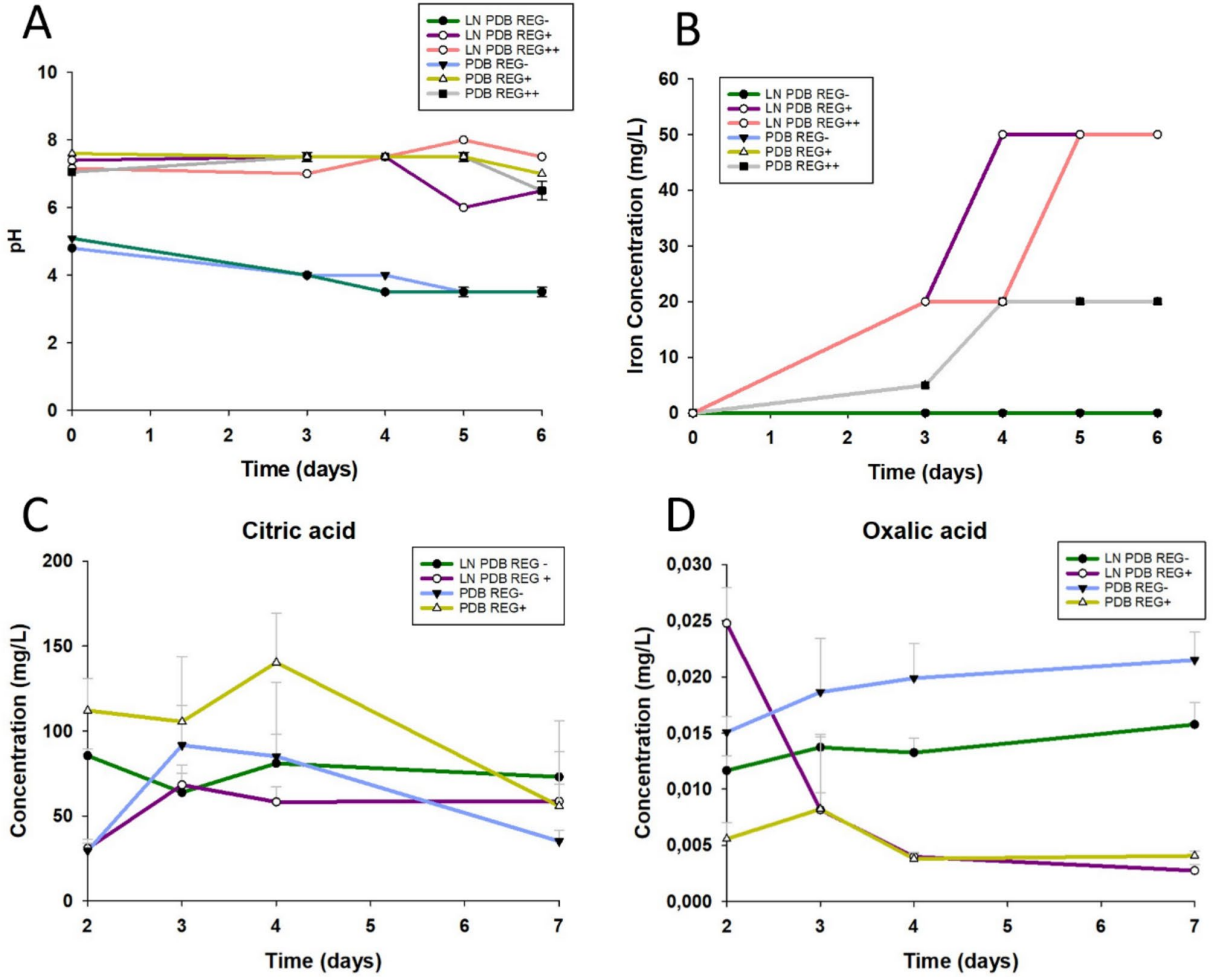
*P. simplicissimum* bioleaching cultures profiling parameters in 20% PDB (Potato Dextrose Broth) (v/v) over 1 week (*n* = 3). **(A)** pH variation on the leachate. **(B)** Iron ions concentration on the leachate. **(C)** Citric acid levels suggesting an initial peak between day 3 and day 4, and the maximum value recorded for the cultures of PDB with 30% EAC-1 A (REG+) (*sd* = 0). **(D)** Oxalic acid levels suggesting an initial peak for cultures supplemented with 30% EAC-1 A (REG+) after which the concentration lowers over time. REG + represents supplementation of the media with EAC-1 A at 30% (w/v). REG + + represents the supplementation of the media with EAC-1 A at 60% (w/v)

To understand how Lunar gravity might impact fungal growth, *P. simplicissimum* was incubated in PDA plates either supplemented with 60% EAC-1 A, or without EAC-1 A (0%), that were exposed to Lunar Simulated Gravity (LSG) on a 2-D Petri-dish Clinostat set to a 10° angle (Fig. 3A). Changes in colony area (Fig. 3B), dried biomass (Fig. 3C), and spore production (Fig. 3D) were measured after 4 days and compared to an Earth gravity control (EG). In EG conditions the supplementation of the medium with EAC-1 A lead to a significant reduction in the colony’s biomass (*p* < 0.001), and spore production (*p* < 0.001), while not significantly affecting the colony area (*p* = 0.064). In colonies grown without EAC-1 A (0%), exposure to Lunar Simulated Gravity (LSG) promoted a 20% increase in colony area (*p* = 0.003) but did not affect biomass (*p* = 0.6) or spore production (*p* = 0.7), when compared to Earth gravity control (EG). In contrast, in colonies grown with 60% Lunar regolith EAC-1 A, exposure to Lunar Simulated Gravity (LSG) did not significantly impact colony area (*p* = 0.062) (Fig. 4B), total biomass (*p* = 0.2) (Fig. 4C), or spore production (*p* = 0.06) when compared to colonies grown in normal gravitational conditions (EG) (Fig. 4D). However, when in Lunar Simulated Gravity (LSG), colonies with EAC-1 A (60%) exhibited 15% less colony area (*p* = 0.008) (Fig. 4B), 46% less biomass (*p* < 0,001) (Fig. 4C), and 21% less spore production (*p* < 0.001) than colonies without EAC-1 A (0%) (Fig. 4D).

### Bioleaching Verification Tests

To validate *P. simplicissimum’s* capacity to mobilize metals from the regolith to the liquid culture medium, bioleaching verification tests were performed in bioreactor flasks by growing fungal cultures in full PDB medium with 60% EAC-1 A regolith for 2-weeks (Supplementary Table 2). Additionally, because both low nutrient availability and presence of toxic metals could trigger changes in the fungus’s bioleaching potential [17, 44], we investigated the impact of using low nutrient (LN) medium (20% PDB), and a reduced amount of EAC-1 A regolith (30%). Culture profiling parameters were monitored to inform the bioleaching process, these were: medium pH, total iron (Fe^3+^ and Fe^2+^) concentration, and levels of organic acid (citric and oxalic acid) (Fig. 4 and Supplementary Tables 3, 4).

**Fig. 5:**
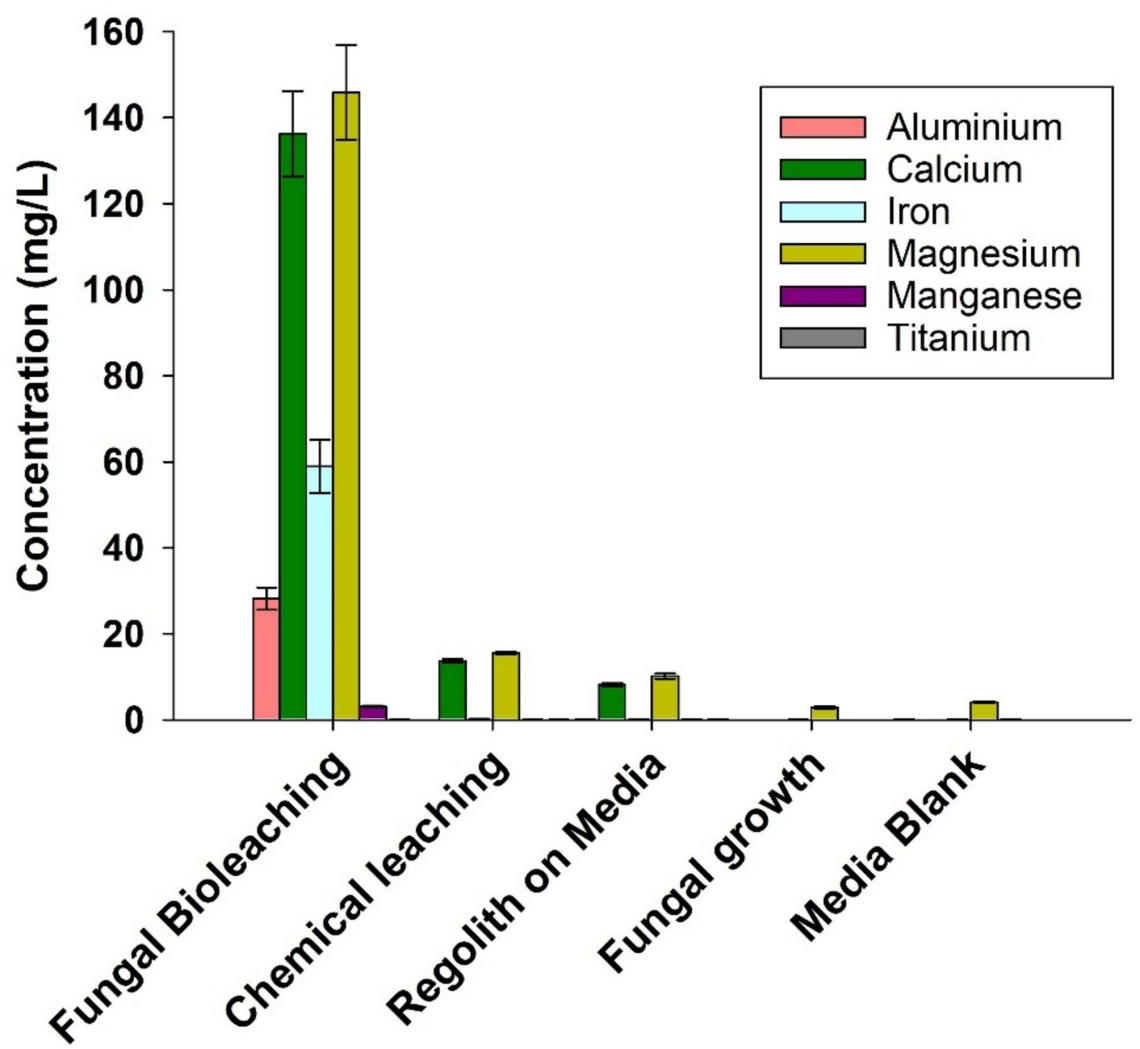
Metallic ion concentrations (mg/L) in the leachate solutions after 2 weeks of fungal bioleaching process using LN media with 30% EAC-1 A (w/v), demonstrating the viability of using the fungus *P. simplicissimum* to extract the metals from Lunar regolith *(n* = 3 per group)

Results show that fungal cultures in full medium without regolith (PDB REG-) reported an acidic pH 4, which remained acidic after 1 week of incubation, registering the lowest pH of 3.5 on day 5 (Fig. 5A). In contrast, the pH remained at 7.5 in full medium cultures with EAC-1 A regolith (PDB REG + and PDB REG++) where the fungal bioleaching process took place (Fig. 5A). Similarly, in bioleaching cultures using a low nutrient medium and a reduced amount of EAC-1 A (LN PDB REG+), the pH profile of the medium ranged from 6 to 7.5 after 1 week of bioleaching, in contrast to the acidic pH 4 of low nutrient medium without EAC-1 A (LN PDB REG-) (Fig. 5A).

Total iron was measured as an indicator of successful metal extraction from the regolith to the leachate solution. Results show that, in fungal cultures without regolith supplementation (REG-), the iron levels in solution remained at 0. In contrast, fungal bioleaching conditions (REG + and REG++) described mobilization of 20 mg/L to 50 mg/L iron from the regolith to the liquid environment by day 6. Additionally, data suggests that reducing nutrients improves bioleaching efficiency, as the experimental conditions using low nutrient medium (LN PDB) reached higher concentrations for iron (50 mg/L) than those using full medium. Furthermore, results show that using a lower volume of EAC-1 A (30%) does not impact the final amount of iron being mobilized, when compared with a higher volume of EAC-1 A (60%) (Fig. 5B and Supplementary Table 4).

Given the key-role of organic acids in the bioleaching process we measured the concentration of both citric and oxalic acid in the bioleaching cultures over 1 week. (Fig. 5C-D). Results indicate that both regolith supplementation and low nutrient medium (LN) induce changes in the levels of organic acids. From all tested conditions, the maximum levels of organic acids were achieved in cultures supplemented with Lunar regolith simulant EAC-1 A during the first 4 days. In fungal bioleaching cultures using full medium with 30% EAC-1 A (PDB REG+), citric acid levels reached a maximum concentration of 140 ± 29 mg/L, which was much higher than the levels achieved in cultures without regolith (91 ± 23 mg/L) (Fig. 4A). Cultures using low nutrient medium accumulated less citric acid than full-medium cultures, registering a maximum of 68 ± 7 mg/L when supplemented with 30% EAC-1 A (at day 3), and 85 ± 4 mg/L in cultures without regolith (at day 2) (Fig. 4A). In contrast, the maximum levels of oxalic acid in the leachate (0.025 ± 0.015 mg/L) were achieved on day 2 in bioleaching cultures using low-nutrient medium with 30% EAC-1 A (LN PDB REG+). In full medium with 30% EAC-1 A (PDB REG+), the peak of oxalic acid occurred only on day 3 (0.008 ± 0.006 mg/L) (Fig. 4D), whereas in fungal cultures without EAC-1 A supplementation, the maximum concentration of oxalic acid occurred only at day 7, in both low nutrient medium (0.016 ± 0.002 mg/L), and in full medium (0.022 ± 0.003 mg/L) (Fig. 4D).

Bioleaching tests demonstrated that a lower nutrient medium and a reduced amount of EAC-1 A regolith would maximize the metals mobilized while reducing the required resources. Therefore, a fungal bioleaching set-up was established using LN medium (20% PDB) supplemented with 30% EAC-1 A (w/v). *P. simplicissimum* bioleaching cultures were grown in bioreactor flasks for 2 weeks, after which the leachate solutions were collected and subjected to analysis using Inductively Coupled Plasma Mass Spectrometry (ICP-MS) to identify and quantify the extracted metals (Fig. 5).

**Fig. 6:**
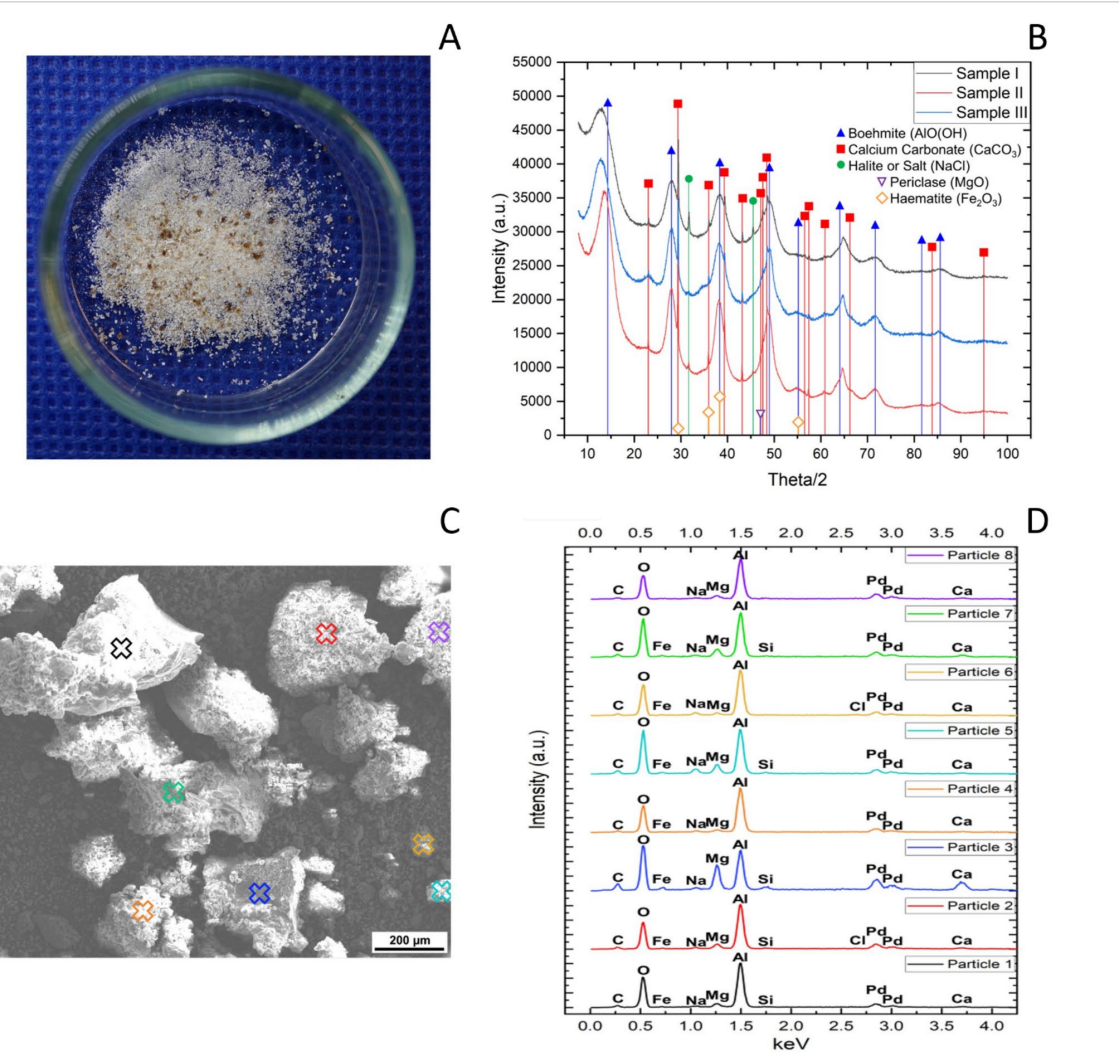
Metals recovered by chemical precipitation from the leachate solutions of the fungal bioleaching cultures of EAC-1 A (*n* = 3). **(A)** Macroscopic view over the mineral powder **(B)** Energy Dispersive X-Ray Spectroscopy analysis of the powder evidencing the presence of metals successfully recovered from the leachate (aluminium, calcium, magnesium and iron) **(C)** Scanning Electron Microscopy of a powder sample, showing heterogeneous particles that differ in size **(D)** X-Ray Diffraction of the mineral powder, detecting metals successfully recovered from the leachate (aluminium, calcium, magnesium and iron)

ICP-MS analysis of the leachate demonstrated the capability of *P. simplicissimum* to extract different metals from EAC-1 A regolith. Results report an efficient extraction of metals during fungal bioleaching, of up to 159 mg/L magnesium, 151 mg/L calcium, 68 mg/L iron, 32 mg/L aluminium, 3 mg/L manganese as well as traces of titanium (0.02 mg/L) (Fig. 6). For comparison, a chemical leaching control (medium + organic acids) was included, which demonstrated successful metal mobilization, however, in much lower amounts than fungal bioleaching cultures (15.5 mg/L of magnesium and 0.2 mg/L of iron). This demonstrates the significance of organic acids, particularly citric and oxalic acid, in mobilizing metals from the regolith particles into the leachate and highlights the use of fungi to maximize the leaching process.

Interestingly, results indicate a baseline magnesium mobilization when using PDB medium (potato-based) for bioleaching, as an initial magnesium concentration of 4 mg/L in medium without regolith increased to 10 mg/L when supplemented with 30% EAC-1 A and incubated for 2 weeks (Supplementary Table 5). It is important to note that PDB-led metal mobilization was not detected for any of the other metals analysed in this study. Results also display the fungus uptake of metal elements, as the concentration of metals dropped after 2 weeks in cultures with *P. simplicissimum* (but without regolith). This happened with magnesium which concentration in the medium was 30% less (3 mg/L) than in flaks containing only PDB (without fungus).

Retrieval of metals from the leachate was successfully achieved, allowing for an extraction of 10.3 ± 3.7 g/L of a metal powder of mostly white color with specs of brown-coloured particles (Fig. 1A). Analysis of the obtained powder via Scanning Electron Microscopy (SEM) shows that heterogeneous particles that differ in size, and subsequent Energy Dispersive X-Ray Spectroscopy analysis (EDS) analysis confirms the presence of aluminum, calcium, magnesium and iron, which validates the recovery process. Interestingly, EDS-analyzed powder fractions show a high abundance of aluminium, with a parallel secondary dominance in either sodium, iron, calcium, or magnesium (Fig. 1B and Supplementary Table 2). This contrasts with the ICP-MS analysis which detected magnesium as the most abundant metal in the leachate solution. Further X-Ray Diffraction (XRD) analysis of the powder (Fig. 1d) reveals a spectrum mostly related to Boehmite (AlO(OH)), with lower amounts of Calcium Carbonate (CaCO_3_) and Halite (NaCl) adding to the broadened aluminium-related peaks. It should be noted that the mineral haematite (Fe_2_O_3_) might also be present in the samples, however signal overlap of the observed peaks could not confirm their detection. The mineral periclase (MgO) was detected, but no Mg-Phases (such as Mg(OH)₂) could be clearly detected in the XRD analysis. Other metals were present in the powder sample, for instance, palladium (Fig. 1d) which signal presence can be traced back to the sputter layer which was applied during sample preparation of SEM/EDS analysis, as well as sodium and chloride, which signals likely result from the precipitation method used to recover the metals.

## Discussion

To become sustainable and cost-effective future missions to the Moon will depend on ISRU technologies such as mining of the Lunar soil [1, 7]. Indeed, the Lunar regolith contains minerals abundant in metallic elements (as shown on Table 1), which are often toxic to microorganisms [45]. Nonetheless, filamentous fungi can tolerate high concentrations of metals, making them suitable candidates for biomining applications [33, 35]. Therefore, in this study, we investigated the ability of the filamentous fungus *P. simplicissimum* to extract metals from the Lunar regolith simulant EAC-1 A. We envisioned a Moon biomining scenario which takes place inside a habitat exposed to Lunar gravity (0.16 g), shielded from radiation, and with an optimal temperature for its occupants (of 22–24 °C, similarly to the ISS).

Initial investigations firmly establish the biocompatibility of the *P. simplicissimum* fungus with EAC-1 A regolith, even at elevated concentrations of up to 60% (Fig. 2). Nevertheless, supplementation with EAC-1 A at 60% lead to reduced spore production and biomass under Earth Gravity (EG) conditions (Fig. 3). When grown under Lunar Simulated Gravity (0.16 g) on a 2-D Petri-dish clinostat, colonies showed a 20% increase in *P. simplicissimum* colony area without EAC-1 A compared to Earth Gravity controls, but no significant changes were detected in biomass or spore production (Fig. 3).

Our bioleaching tests report the ability of *P. simplicissimum* to efficiently mobilize iron (up to 50 mg/L) using low nutrient medium with a reduced amount of EAC-1 A regolith. This is particularly important as the medium chosen is based on potato infusions and sugar (which is cost-effective, non-toxic, and can possibly be obtained from potatoes grown on board) allowing for future fungal bioleaching approaches to minimize the use of resources. We furthermore report a successful fungal bioleaching of EAC-1 A regolith by the fungus *P. simplicissimum*, using 40 mL bioreactor flasks with low nutrient medium (20% PDB) and 30% EAC-1 A (w/v), which results in the extraction of magnesium (159 mg/L), calcium (151 mg/L), iron (68 mg/L), aluminium (32 mg/L), manganese (3 mg/L) and titanium (0.02 mg/L) in the leachate solution, after 2 weeks at room temperature (22–24 °C). Moreover, we report the successful metal recovery from the leachate solution to a promising average of 10 ± 3 g/L of metal oxide powder. Further SEM/EDS and XRD analysis confirm the high abundance of aluminium [as boehmite (AlO(OH))], magnesium [possibly as trace amounts of periclase (MgO) from the regolith], and iron [possibly as haematite (Fe_2_O_3_)] and magnetite [possibly as (Fe_3_O_4_)] in the recovered metal oxide powder. The differences in metal measurements in the leachate (by ICP-MS) and in the metal oxide powder weighed after precipitation (by EDS and XRD) are directly related to the precipitation methodology itself and can result from various factors. For instance, the weight recorded after precipitation does not correspond to the metallic ions alone but rather to the minerals containing them, as well as the addition (i.e. sodium from NaOH) and removal of elements from the original leachate. Moreover, only MgO (and not MgOH_2_) was detected the final powder, likely as direct result from the precipitation method used in this study, as MgOH_2_ is soluble at high pH, and also because the XRD analysis could not clearly detected Mg-Phases. Additionally, as measured by the XRD analysis, it is possible to identify traces of carbon molecules directly linked to the fungus metabolism. The minerals extracted can be further purified and refined to be used in technology production for construction materials or power generation devices [1, 12]. For instance, the extracted magnesium (159 mg/L) is a metal whose alloys are known for their light structure and is therefore highly attractive for space exploration, where increased weight of equipment always requires the use of more fuel [46, 47]. In turn, usage of an *in situ-*produced aluminium could be included in the manufacture of space vehicles, and solar cells [48, 49]. Moreover, novel research has shown that biomined iron has potential for use in synthesizing components (replacement parts such as screws) that could integrate systems for space exploration [31]. Therefore, metals extracted via fungal bioleaching can aid future Lunar habitats in becoming independent from Earth and possibly create avenues for commercial contributions [4].

Biomining of lunar regolith simulant has also been demonstrated by Kaksonen et al. using acidophilic bacteria *Acidithiobacillus ferrooxidans*, resulting in successful mobilization of metals such as magnesium (~ 300 mg/L) and aluminium (~ 60 mg/L), after 20 days incubation [30]. However, the study used a different regolith simulant − 1% Lunar Mare Simulant (LMS-1) – which was supplemented with 12.5 mM Fe. LMS-1 is primarily composed of approximately 42.81 wt% SiO₂, 4.62 wt% TiO₂, 14.13 wt% Al₂O₃, 7.87 wt% FeO, 18.89 wt% MgO, and other minor oxides, mimicking the basaltic composition of lunar mare regions [2, 30, 50], whereas the current study used EAC-1 A lunar regolith simulant, which is more similar to the Lunar regolith obtained from Apollo 17 samples [9]. Interestingly, the driving biomining mechanism described for *A. ferrooxidans* was acidolysis – where the low pH of the cultures leads to the mobilization of the metals into solution.

In contrast, in our study fungal bioleaching of EAC-1 A seemed to have occurred mainly at an almost neutral pH (7.5). Moreover, our results show that the peak accumulation of organic acids in the leachate did not correlate with the minimum pH measurements, or with the maximum amount of metals mobilized, challenging the assumption that acidolysis is the main driving mechanism for fungal bioleaching of EAC-1 A regolith [51]. Instead, the fungal bioleaching likely involved additional mechanisms beyond bulk acidification. Studies have shown that fungi can induce localized acidification at the hypha-mineral interface, facilitating the mobilization of the metals at micro-scales, even in environments where the bulk pH remains neutral [52, 53]. Furthermore, direct fungal interactions, such as biomechanical weathering and secretion of chelating compounds, may also play a role in the bioleaching process [52]. Further research is needed to unravel the specific driving mechanisms of *P. simplicissimum*-mediated leaching of EAC-1 A.

While our study is limited in studying leaching mechanisms, the XRD analysis of the final metal powder obtained reported a broad peak for boehmite (a mineral containing aluminium), which suggests a reaction of the metal with secondary metabolites. This, in turn, may indicate the reaction with a complexing agent [54]. Thus, one possible mechanism at play in fungal bioleaching of EAC-1 A is complexolysis – when chelating agents react with the metals, forming either soluble or precipitated complexes that help stabilize the metal ions from the regolith [55, 56], reducing their bioavailability and therefore also reducing metal toxicity toward the fungus [57]. Other bioleaching processes might also be involved in fungal bioleaching of EAC-1 A, for instance: redoxolysis, where enzymatic reactions modify the oxidation state of the metal, enhancing its solubility [58]; and/or bioaccumulation, where metal ions can be assimilated into living cells, either in vacuoles or the cell wall [59]. Moreover, the size of the regolith strongly influences bioleaching efficiency, as smaller particles provide a greater area for dissolution, thereby enhancing metal solubilization and extraction rates [60]. For example, industrial applications often require smaller pellets due to rheological (outflow) problems [61]. From a biological point of view, it is important to note that a filamentous fungus, such as *P. simplicissimum*, has a vast metabolome. This indicates that *P. simplicissimum* has the capacity to produce various other organic acids (such as gluconic acid) and an array of other metabolites [62]. Such compounds may have an impact on the bioleaching potential, especially those with chemical groups such as the carbonyl, amino, hydroxyl and nitro groups, which can interact with the metals on the regolith [63,64]. Although our data cannot confirm the presence of metal oxalates, during fungal bioleaching the metals reaction with complexing agents can lead to the formation of metal oxalates, such as calcium oxalate or magnesium oxalate. These metal oxalates have low solubility at alkaline pH and can lead to the precipitation of chelated metals [65–67]. Further studies are needed to evaluate the mechanisms of fungal bioleaching in detail, including the role of specific metabolites and how they interact with the metal ions in the regolith.

Our study demonstrates fungal bioleaching as a promising ISRU technology in Lunar missions, however, the application of fungal biomining in a real space habitat will require a deeper understanding of the bioleaching mechanisms, as well as process optimizations and modifications. For instance, future fungal bioleaching setups should accommodate both downscaling, for miniaturized testing at Low Earth Orbit (e.g. aboard the ISS), as well as upscaling, for high-volume testing of technologies (e.g. ground-based facilities such as the ESA-DLR LUNA habitat) [12]. Another critical aspect would be to develop a medium with fewer nutrients, and fewer lunar regolith, without compromising the metal mobilization of the tested fungal bioleaching setup. Small-scale biomining has been recently demonstrated on board the ISS with bacteria extracting rare earth elements under different gravitational regimes, and from different regolith analogues [28, 32]. Additionally, recent efforts have been made to develop a novel miniaturized space biomining reactor to conduct metal leaching with the bacterial strains *S. desiccabilis*,* B. subtilis* and *Cupriavidus metallidurans* [28, 32]. Apart from the upcoming Bioasteroid mission aboard the ISS, that will test filamentous fungi potential to mobilize metal onboard the ISS [14], to our knowledge there are no studies addressing fungal biomining in a space-relevant scenario, highlighting the importance of our study (Table 2).

## Conclusion

Our study marks an important first step in the utilization of fungal bioleaching as a promising ISRU technology in future space exploration missions to the Moon. We demonstrate the biocompatibility of *P. simplicissimum* to high concentrations of EAC-1 A regolith (up to 60%), both on Earth gravity and Lunar simulated gravity via clinorotation. We report the successful fungal bioleaching of EAC-1 A regolith by the fungus *P. simplicissimum*, using 40 mL bioreactor flasks with low nutrient medium (20% PDB) and 30% EAC-1 A (w/v), which resulted in the extraction of magnesium (159 mg/L), calcium (151 mg/L), iron (68 mg/L), aluminium (32 mg/L), manganese (3 mg/L) and titanium (0.02 mg/L) in the leachate solution, after 2 weeks at room temperature (22–24 °C). And we report the successful metal recovery from the leachate solution to a promising average of 10 ± 3 g/L of metal oxide powder. Further SEM/EDS and XRD analysis confirm the high abundance of aluminium [as boehmite (AlO(OH))], magnesium [possibly as periclase (MgO)], and iron [possibly as haematite (Fe_2_O_3_)] and magnetite [possibly as (Fe_3_O_4_)] in the recovered metal oxide powder.

Most importantly, we emphasize the use of fungal biomining in space exploration scenario, as it offers several advantages. Namely, fungi are natural cell-factories, with highly resistant spores that are easy to dry-store and can endure space radiation [25]. In addition, fungal biomining is non-exclusive, allowing the selective precipitation of specific metals according to the crew’s necessities [14, 68]; and, given the wide span of fungal biotechnological applications [23], fungal biomining can be easily paired with other processes within the space habitat, for example, with bioremediation of soil for agriculture [67], or electronic waste-recycling [64, 68–71]. Besides, the versatility of biomining as ISRU means it can be applied further beyond the Lunar regolith [14, 28, 71]. Similarly, we envision the utilization of fungal biotechnology as a crucial tool to achieve sustainability and circular economy, both on Earth and in space [71].

## Electronic supplementary material

Below is the link to the electronic supplementary material.

Supplementary Material 1

## Data Availability

All data generated during this study are included in this published article and its supplementary information files. No additional data were generated or analysed.
