# Supplementary material for: Biomining of lunar regolith simulant EAC-1 A with the fungus Penicillium simplicissimum
Source: Fungal Biol Biotechnol. 2025 May 19;12:8. doi: 10.1186/s40694-025-00201-z (PMC12087194; doi:10.1186/s40694-025-00201-z)
Supplement: Supplementary file 1 — Supplementary Material 1 [file 40694_2025_201_MOESM1_ESM.docx]

**Supplementary Table S1.** Impact of simulated Lunar gravity (LSG) compared to Ground orbital shaking (60 rpm), and Ground static conditions, on *P. simplicissimum* growth parameters (colony area and spore production) when grown in PDA (Potato Dextrose) agar plates with and without 60% EAC-1A over 4 days.

|  | **Clinostat**  **60% EAC-1A** | **Clinostat**  **0% EAC-1A** | **Ground**  **60% EAC-1A** | **Ground**  **0% EAC-1A** |
| --- | --- | --- | --- | --- |
| Colony Area (cm^2^) | 4.99 ± 0.13 | 5.23 ± 0.11 | 3.47 ± 0.11 | 4.56 ± 0.19 |
| Spores per mL | 4.8E^5^ ± 8.00E^4^ | 5.9E^5^ ± 1.0E^5^ | 5.40E^5^ ± 1.50E^5^ | 6.20E^5^ ± 1.30E^5^ |

**Supplementary Table S2.** Semi-quantitative detection of metal ions in solution after 2 weeks, showing clear evidence of the fungus capacity to mobilize metallic ions from the regolith to the liquid media.

|  | **PDB with**  **60 % EAC-1A** | **PDB without**  **regolith** | **Fungal**  **Bioleaching** |
| --- | --- | --- | --- |
| Iron | + | + | ++ |
| Aluminium | + | - | ++++ |
| Calcium | + | + | +++ |

– 0 mg/L
+ values between 0 mg/L and 25 mg/L
++ values between 25 mg/L and 100 mg/L,
+++ values between 100 mg/L and 500 mg/L
++++ values higher than 500 mg/L

**Supplemental Table 3.** pH measurements, (with standard error), of the leachates on the tested cultures over 1 week, at 150 rpm 22 °C.

| **TIME** | **PDB**  **0% EAC-1A** | **LN**  **0% EAC-1A** | **PDB**  **30% EAC-1A** | **LN**  **30% EAC-1A** | **PDB**  **60% EAC-1A** | **LN**  **60% EAC-1A** |
| --- | --- | --- | --- | --- | --- | --- |
| day 3 | 4.0 ± 0.0 | 4.0 ± 0.0 | 7.5 ± 0.0 | 6.0 ± 0.0 | 7.5 ± 0.0 | 7.0 ± 0.1 |
| day 4 | 4.0 ± 0.0 | 3.5 ± 0.0 | 7.5 ± 0.0 | 7.5 ± 0.0 | 7.5 ± 0.0 | 7.5 ± 0.0 |
| day 5 | 3.5 ± 0.1 | 3.5 ± 0.0 | 6.0 ± 0.0 | 7.5 ± 0.0 | 7.5 ± 0.1 | 8.0 ± 0.0 |
| day 6 | 3.5 ± 0.0 | 3.5 ± 0.0 | 6.5 ± 0.1 | 7.5 ± 0.0 | 7.0 ± 0.3 | 7.5 ± 0.0 |

**Supplemental Table 4.** Semi-quantitative detection of total metal ions (Fe_2+_ and Fe_3+_) in mg/L on the leachates of the tested cultures over 1 week at 150 rpm 22 ºC.

| **TIME** | **PDB**  **0% EAC-1A** | **LN**  **0% EAC-1A** | **PDB**  **30% EAC-1A** | **LN**  **30% EAC-1A** | **PDB**  **60% EAC-1A** | **LN**  **60% EAC-1A** |
| --- | --- | --- | --- | --- | --- | --- |
| day 3 | 0 | 0 | 5 | 20 | 5 | 20 |
| day 4 | 0 | 0 | 20 | 50 | 20 | 20 |
| day 5 | 0 | 0 | 20 | 50 | 20 | 50 |
| day 6 | 0 | 0 | 20 | 50 | 20 | 50 |

**Supplemental Table 5.** Concentration of metallic ions, in mg/L, in the leachate solution of EAC-1A bioleaching cultures, and corresponding controls after 2 weeks of incubation at room temperature (22 - 24°C).

|  | **Al** | **Ca** | **Fe** | **Mg** | **Mn** | **Ti** |
| --- | --- | --- | --- | --- | --- | --- |
| **FUNGAL BIOLEACHING** | 28.17 ± 2.62 | 136.23 ± 9.91 | 58.89 ± 6.21 | 145.83 ± 11.02 | 3.08 ± 0.16 | 0.02 ± 0.001 |
| **CHEMICAL LEACHING** | 0.06 ± 0.0 | 13.67 ± 0.42. | 0.18 ± 0.001 | 15.53 ± 0.29 | 0.01 ± 0.0002 | 0.01 ± 0.0005 |
| **REGOLITH ON MEDIA** | 0.05 ± 0.01 | 8.13 ± 0.44 | 0.090 ± 0.01 | 10.13 ± 0.58 | 0.00 ± 0.003 | 0.01 ± 0.0002 |
| **FUNGAL GROWTH** | 0.01 ± 0.0 | 0.03 ± 0.0 | 0.00 ± 0.0008 | 2.80 ± 0.27 | 0.00 ± 0.0 | 0.00 ± 0.0 |
| **MEDIA BLANK** | 0.05 ± 0.05 | 0.03 ± 0.0 | 0.02 ± 0.003 | 4.07 ± 0.16 | 0.00 ± 0.0 | 0.00 ± 0.0 |
